# Cyto-Genotoxic and Behavioral Effects of Flubendiamide in *Allium cepa* Root Cells, *Drosophila melanogaster* and Molecular Docking Studies

**DOI:** 10.3390/ijms24021565

**Published:** 2023-01-13

**Authors:** İbrahim Hakkı Ciğerci, Recep Liman, Erman Salih İstifli, Dilek Akyıl, Arzu Özkara, Elena Bonciu, Florica Colă

**Affiliations:** 1 Department of Molecular Biology and Genetics, Faculty of Science and Literature, Afyon Kocatepe University, 03200 Afyonkarahisar, Turkey; 2 Department of Molecular Biology and Genetics, Faculty of Arts and Sciences, Uşak University, 1 Eylül Campus, 64300 Uşak, Turkey; 3 Department of Biology, Faculty of Science and Literature, Cukurova University, 01330 Adana, Turkey; 4 Department of Agricultural and Forestry Technology, Faculty of Agronomy, University of Craiova, 13 A.I. Cuza Street, 200585 Craiova, Romania

**Keywords:** chromosome aberrations, DNA damage, environmental risk assessment, pesticides, toxicity

## Abstract

Flubendiamide (FLB) is an insecticide that is commonly employed to control pests on a variety of vegetables and fruits, with low toxicity for non-target organisms. However, due to its widespread use, the environmental risks and food safety have become major concerns. In this study, the toxicity potential of FLB was studied in the model organisms, *Allium cepa* and *Drosophila melanogaster*. The cyto-genotoxic effects of FLB on the root growth, mitotic index (MI), chromosomal aberrations (CAs) and deoxyribonucleic acid (DNA) damage in *A. cepa* root meristematic cells were investigated using the root growth inhibition *Allium* test and Comet assays. FLB caused CAs in the form of disturbed ana-telophase, chromosome laggards, stickiness, anaphase-bridge and polyploidy depending on the concentration and the exposure time. The toxicity and genotoxicity of FLB at various doses (0.001, 0.01, 0.1 and 1 mM) on *D. melanogaster* were investigated from the point of view of larval weight and movement, pupal formation success, pupal position, emergence success and DNA damage, respectively. FLB exposure led to a significant reduction of the locomotor activity at the highest concentration. While DNA damage increased significantly in the FLB-treated onions depending on the concentration and time, DNA damage in the FLB-treated *D. melanogaster* significantly increased only at the highest dose compared to that which occurred in the control group. Moreover, to provide a mechanistic insight into the genotoxic and locomotion-disrupting effects of FLB, molecular docking simulations of this pesticide were performed against the DNA and diamondback moth (DBM) ryanodine receptor (RyR) *Repeat34* domain. The docking studies revealed that FLB binds strongly to a DNA region that is rich in cytosine-guanine-adenine bases (C-G-A) in the minor groove, and it displayed a remarkable binding affinity against the DBM RyR *Repeat34* domain.

## 1. Introduction

Pesticides are common agricultural chemicals used to control pests, and thereby, increase the crop yield. Their uses are not limited to the agricultural field, but they are also used to control mosquitoes, mice and harmful insects in houses and workplaces [1,2]. For this reason, the investigation of the negative effects of these substances, which are abundant in the surrounding environment, on off-target organisms is among the popular topics of today’s modern life.

Flubendiamide, FLB, which belongs to the phthalic acid diamide class of pesticides, is presented as a white crystalline powder, with the chemical formula, C_23_H_22_F_7_IN_2_O_4_S. The molecular weight of FLB is 682.4 g·mol^−1^, its density is 1.659 g·cm^−3^, and its melting point is within the range of 217.5–220.7 °C [3]. FLB is an insecticide that is widely used to control pests on cotton, rice, some vegetables, okra, tobacco, corn and fruits, and it is argued to have low toxicity to non-target organisms [4,5]. As an orally ingested toxicant, the mode of action of FLB is larvicidal activity. This results in the rapid cessation of feeding and extended residual control [5]. However, due to the widespread use of FLB, the environmental risks and food safety have become major concerns. Therefore, it is important to develop fast and practical methods for the detection of residual FLB in the environment [2]. Although it is known to have a low toxicity potential, it has been shown in recent years that it causes morphological anomalies and neurotoxic effects on different non-target organisms [5]. Thus, the toxic effect of FLB, a widely used pesticide, in terms of its potential environmental risks, needs to be clarified using different test systems.

Many different international organizations, including the World Health Organization (WHO), have recommended higher plant models for the evaluation of the genotoxicity of various chemical substances [6,7]. Plant chromosomes are excellent biomarkers for mutagenicity studies, and they can be used in the biomonitoring of various environmental pollutants because they are useful, reliable and economical [8]. The *Allium* test for higher plants is a highly sensitive, reliable, easy-to-apply, economical and rapid test that is often used to detect environmental genotoxins, mutagens and pesticides [9,10,11,12]. Using this test, a comprehensive mutagenic evaluation of different chemical compounds, phytochemicals, nanomaterials and different environmental samples can be achieved successfully [13,14]. The *Allium* test is a widely used technique that not only evaluates the cytotoxicity of various chemicals, but it also to determine the genotoxicity with the application of the highly sensitive comet test.

In connection with the comet assay, behavioral toxicity experiments are also carried out based on larval weight and movement, pupa formation success, adult weight, exit success, pupal position, negative geotaxis and longevity to evaluate the effects of different chemicals on *D. melanogaster* [15].

*D. melanogaster* is one of the model organisms used in toxicity studies of chemical substances with many different properties. Although they differ significantly from vertebrates in appearance, the genes and neural circuits controlling basic behaviors are functionally conserved during evolution. Furthermore, the *Drosophila* species are well suited for constructing disease models that can largely recapitulate the pathological states and behavioral changes in humans and for developing potential therapeutic drug targets, making them ideal for studying the genetic and circuitry underpinnings of normal behavior. For this reason, the *Drosophila* species is a preferred model organism in many studies [16]. In recent years, the in vivo comet assay has gained importance in the evaluation and detection of the DNA-damaging and genotoxic effects of different chemical substances. This assay, also known as single-cell gel electrophoresis (SCGE), is a preferred method for the evaluation of the potential genotoxicity of many chemicals due to its simplicity, versatility, sensitivity, reliability and relatively low cost [17]. At the same time, the comet assay is a popular test system that can be applied to many different organisms or cells.

Molecular docking has become a widely used bioinformatics technique in recent years to determine the genotoxic mode of action or to find unknown binding sites of ligands on specific receptors, which greatly assists in elucidating the ultimate biological effect [18,19,20,21,22]. In this context, although there have been a few experimental studies of FLB on genotoxicity and behavior in various non-target organisms, there is still no molecular-level explanation for the structural mechanistic reasons for these observed biological effects [23,24,25]. Therefore, considering the toxic effects of FLB in the non-target organisms described above, this study was designed to reveal the cyto-genotoxic, behavioral and neurotoxic effects of FLB in two different eukaryotic test organisms. Within the scope of the study, the potential genotoxicity of FLB, an insecticide widely used in agriculture in recent years, was evaluated with different test systems by following a multi-faceted approach. In this study, the cyto-genotoxic effects of FLB on two different eukaryotic organisms were elucidated by the examination of the mitosis (mitotic index), root growth (root growth inhibition), induced chromosomal abnormalities and DNA damage (comet assay) in *Allium cepa*, in combination with the investigation of the behavioral toxicity and DNA damage (comet assay) response in *D. melanogaster*. Furthermore, possible receptor-ligand interactions, which provide a mechanistic perspective on the genotoxic and behavior-disrupting effects of FLB, were elucidated for the first time by a molecular docking technique, in which the DNA and diamondback moth (DBM) ryanodine receptor (RyR) was used as a target (receptor) molecule.

## 2. Results

### 2.1. Allium Test, D. melanogaster Behavioral Assay and Comet Assay

**Figure 1:**
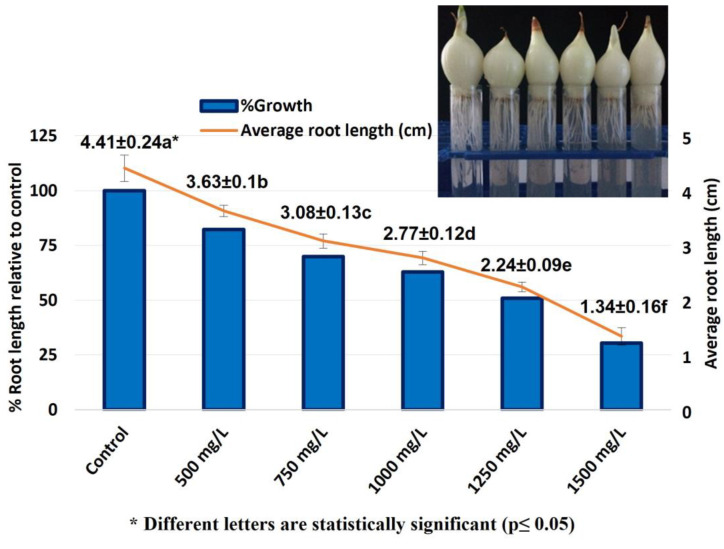
The inhibitory effect of FLB on *A. cepa* root growth. r = −0.973; *p* = 0.01.

In this study, the cyto-genotoxic effects of FLB on the root growth, mitotic index (MI), chromosome aberrations (CAs) and DNA damage in *A. cepa* root meristematic cells were investigated using the *Allium* ana-telophase test. In the *Allium* root growth inhibition test, the EC_50_ concentration was determined prior to the experiments using five different FLB concentrations: 500, 750, 1000, 1250 and 1500 mg/L (Figure 1).

The concentration value of FLB (EC_50_), which reduced the root growth by 50% compared with that which occurred in the negative control group, was determined as being 1250 mg/L. Therefore, *A. cepa* roots were exposed to three different concentrations of FLB (625, 1250 and 2500 mg/L, as 1/2 × EC_50_, EC_50_ and 2 × EC_50_ of the determined EC_50_), alongside to distilled water (negative control) and methyl methane sulfonate (MMS, 10 ppm, positive control) for 24, 48, 72 and 96 h.

The effect of FLB on the MI and mitotic phases in the *A. cepa* root cells is shown in Table 1. According to the *Allium* test results, all of the applied FLB concentrations significantly reduced the MI compared to that which occurred in the control group (F = 189.8, df = 4, *p* = 0.00 for 24 h; F = 358.4, df = 4, *p* = 0.00 for 48 h; F = 314.4, df = 4, *p* = 0.00 for 72 h; F = 544.1, df = 4, *p* = 0.00 for 96 h).

**Figure 2:**
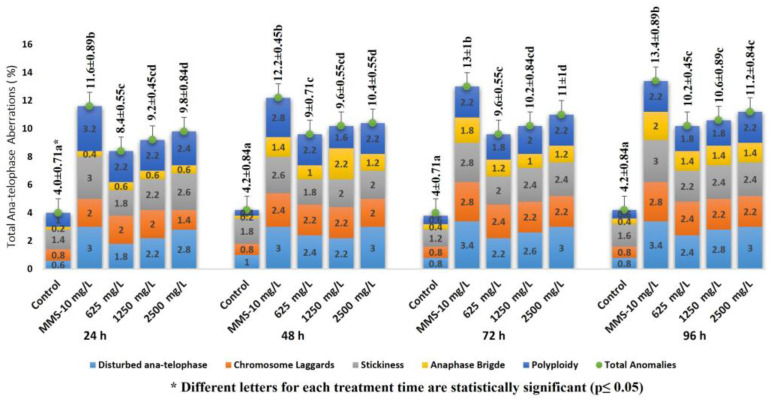
The variation of the total number of CAs in *A. cepa* root cells depending on FLB concentrations and time exposure. Dose dependent: For 24 h, r = 0.71; *p* = 0.01. For 48 h, r = 0.725; *p* = 0.01. For 72 h, r = 0.616; *p* = 0.05. For 96 h, r = 0.518; *p* = 0.05. Time dependent: For 625 mg/L, r = 0.635; *p* = 0.01. For 1250 mg/L, r = 0.646; *p* = 0.01. For 2500 mg/L, r = 0.586; *p* = 0.01.

**Figure 3:**
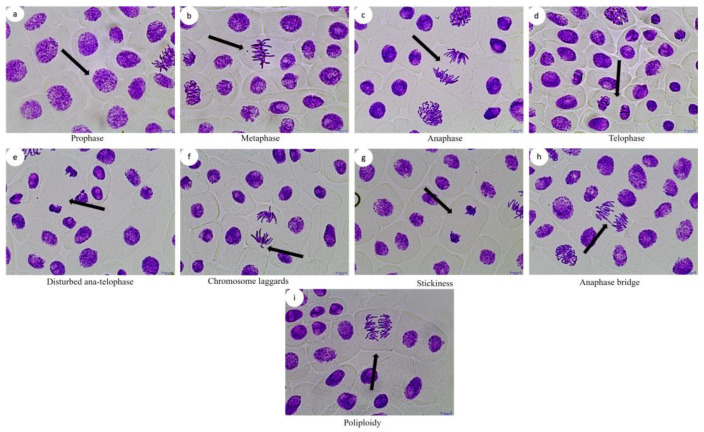
Typical stages of mitosis (**a**–**d**) and ana-telophase anomalies (**e**–**i**) induced by FLB in *A. cepa* cells. Scale bars: 10 µm.

The percentage changes in the total CAs as a result of the FLB concentrations and time exposure were found to be statistically significant compared to those of the control (F = 80, df = 4, *p* = 0.00 for 24 h; F = 111.1, df = 4, *p* = 0.00 for 48 h; F = 80.7, df = 4, *p* = 0.00 for 72 h; F = 91.81, df = 4, *p* = 0.00 for 96 h). Therefore, the total number of CAs was found to be both concentration- and time dependent (Figure 2). It was determined that most of the CAs induced by FLB in the *A. cepa* root cells were the following types: disturbed ana-telophase, chromosome laggards, stickiness, anaphase bridge and polyploidy (Figure 3).

**Figure 4:**
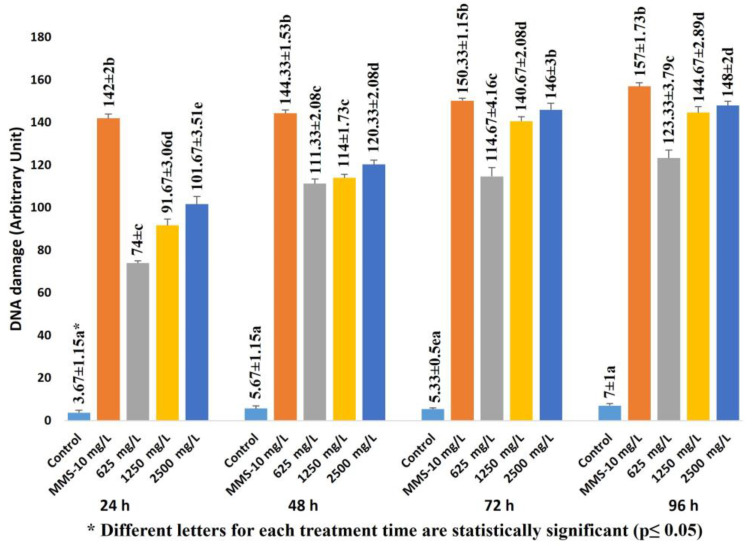
DNA damage induced by FLB in *A. cepa* roots. Dose dependent: For 24 h, r = 0.969; *p* = 0.01. For 48 h, r = 0.895; *p* = 0.01. For 72 h, r = 0.918; *p* = 0.01. For 96 h, r = 0.900; *p* = 0.01. Time dependent: For 625 mg/L, r = 0.888; *p* = 0.01. For 1250 mg/L, r = 0.962; *p* = 0.01. For 2500 mg/L, r = 0.953; *p* = 0.01.

The results from the comet assay are summarized in Figure 4. The MI results and CAs correlate well with that of the comet assay. As can be seen, exposure to FLB increased the DNA damage rate at all concentrations in a dose- and time-dependent manner. The comet assay results showed significantly higher DNA damage values at all of the FLB concentrations compared to those of the negative control (F = 1265.8 df = 4 *p* = 0.00 for 24 h; F = 2836.5, df = 4, *p* = 0.00 for 48 h; F = 1720, df = 4, *p* = 0.00 for 72 h; F = 2059.5, df = 4, *p* = 0.00 for 96 h).

**Figure 5:**
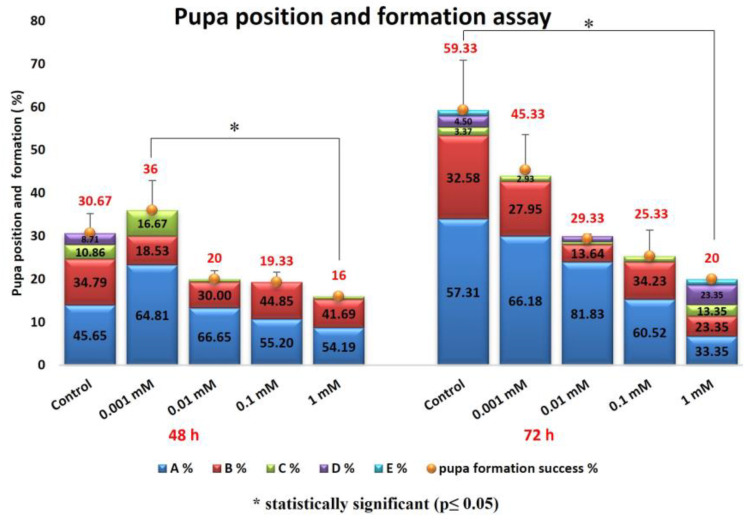
Effect of FLB exposure on pupa position and formation success of *D. melanogaster*.

**Figure 6:**
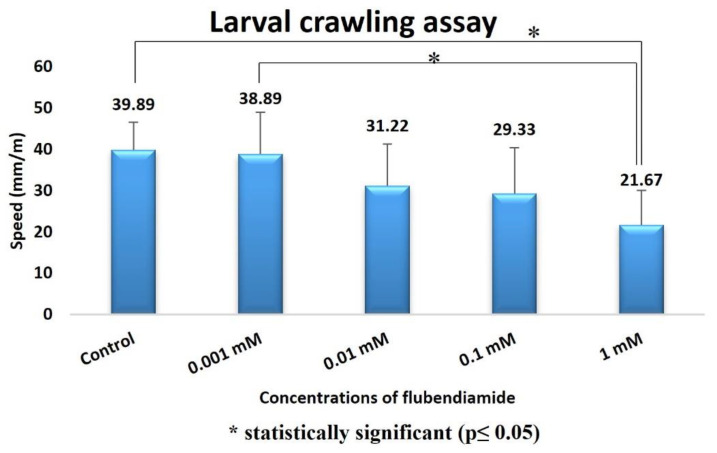
Effect of FLB exposure on larval crawling of *D. melanogaster*.

The effects of the 48 and 72 h treatments with FLB on pupal formation success are provided in Figure 5. It can be observed that the FLB concentration increase negatively affected the success of the pupa formation. There was a statistically significant difference between the 0.001 mM and 1 mM groups in the 48 h administration test (H = 3.14; df = 1; *p* = 0.017) and between the control group and 1 mM administration group at 72 h (H = 2.99; df = 1; *p* = 0.027). The results obtained in the pupal position experiment with FLB showed us that there may be an interaction between this substance and the DNA (Figure 5). According to the data we obtained in the larval crawling assay (Figure 6), a decrease in larval movement was observed on the samples due to the increase in the FLB concentration. The reduction of larval crawling was found to be statistically significant between the 1 mM and 0.001 mM groups (H = 3.22; df = 1; *p* = 0.012) and the 0.001 mM and control groups (H = 3.30; df = 1; *p* = 0.01).

**Figure 7:**
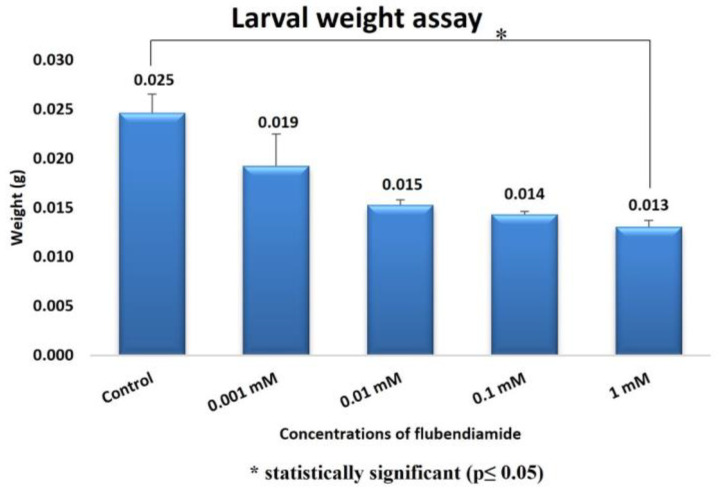
Effect of FLB exposure on larval weight of *D. melanogaster*.

In our study, the larval weight (Figure 7) reduction was found to be statistically significant between the control group and the 1 mM group (Mann–Whitney U = 3.19; df = 1; *p* = 0.014). 

**Figure 8:**
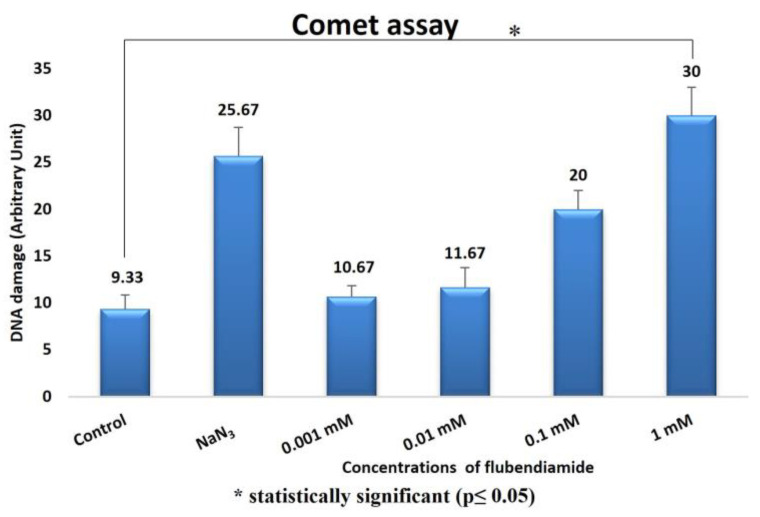
FLB-induced DNA damage scores in *D. melanogaster*.

The comet assay results in *D. melanogaster* are shown in Figure 8. In this respect, an increase in the amount of DNA damage was observed depending on the administration of increased FLB concentrations. The increase in the DNA damage scores between the control group and the 1 mM FLB-treated group was statistically significant (H = 3.1; df = 1; *p* = 0.029).

### 2.2. DNA–FLB Molecular Interactions

**Figure 9:**
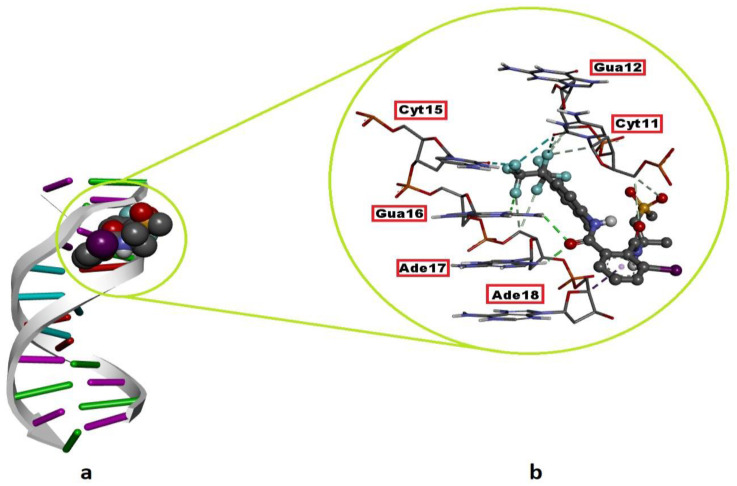
Top-ranked conformation of the intermolecular interaction between flubendiamide and B-DNA. (**a**) Cartoon view of the DNA–flubendiamide complex; (**b**) 3D DNA-ligand interaction diagram of the top-ranked docking conformation. Nucleotides in interaction with flubendiamide are outlined in red boxes. The green and light grey dashed lines on the right image represent hydrogen bonds, whereas the purple and light cyan dashed lines represent the hydrophobic contact and halogen (fluorine) interactions, respectively.

The DNA nucleotides interact with FLB, the binding mode, and the types of resulting intermolecular interactions are given in Table 2. FLB showed its best binding mode inside the minor groove of the B-DNA mediated by Gua12, Cyt15, Gua16, Ade17 and Ade18. The snug fit of FLB in this region of the DNA molecule is stabilized by H bonds (Cyt11, Gua12, Gua16 and Ade17), hydrophobic contacts (Ade18) and halogen (fluorine) interactions (Cyt11 and Cyt15) (Figure 9; Table 2). The binding strength (free energy of binding) between the FLB and B-DNA was remarkably strong (ΔG_best_ = −7.25) when it was compared with those of the experimental DNA mutagens, MMS (ΔG_best_= − 4.07) and NaN_3_ (ΔG_best_= −4.13). Although FLB in its most favorable conformation did not bind to complementary nucleotides in the DNA, it showed the ability to bind with both strands of the DNA and showed the same mode of binding (minor groove recognition) as the mutagens MMS and NaN_3_ did, which may indicate the DNA-reactive mutagen property of this agent (Figure 9; Table 2).

### 2.3. Intermolecular Interactions between FLB and Diamondback Moth (DBM) RyR Repeat34 Domain

**Figure 10:**
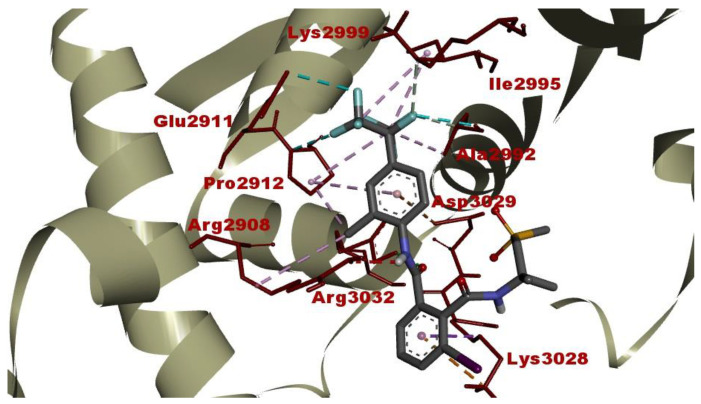
Post-docking top-ranked conformation of flubendiamide in complex with DBM RyR *Repeat34* domain. The green and light grey dashed lines represent hydrogen bonds, whereas the purple and light cyan dashed lines represent hydrophobic contacts and halogen (fluorine) interactions, respectively.

The resulting intermolecular interactions and binding affinity of FLB against the DBM RyR *Repeat34* domain are provided in Table 3 and Figure 10. 

The interactions of FLB within the glycerol-binding pocket of the DBM RyR *Repeat34* domain consist of three H bonds with Arg2908, Pro2912, Ala2992, Lys2999 and Arg3032, several hydrophobic alkyl and π-interactions (π-sigma and π-alkyl) with the residues Arg2908, Pro2912, Ala2992, Ile2995 and Lys3028, two electrostatic interactions with Lys3028 and Asp3029 and three halogens (fluorine) interactions with Glu2911, Pro2912 and Ala2992, respectively (Table 3; Figure 10).

Compared with the native ligand (glycerol), FLB exhibited a very strong and favorable binding affinity (ΔG_best_= −8.23 kcal/mol) against the DBM RyR *Repeat34* domain, where the glycerol showed a weak-to-moderate binding affinity of −3.32 kcal/mol (Table 3). When it was compared in terms of the calculated inhibition constant (Ki) (data not given), the binding capacity of FLB with the DBM RyR *Repeat34* domain is approximately 4140 times that of glycerol. This difference reflects the extent to which the FLB can stabilize the RyR receptor in its ‘open conformation’.

## 3. Discussion

The increase in the use of heavy metals and chemical agents, together with the developing technological advances, and the increase in the use of intensive pesticides and fertilizers in agricultural applications has led to increased exposure to all of these chemical substances. This situation poses a risk in terms of the effects of these chemicals on non-target organisms, and it is important to analyze these risks in terms of the acute or chronic effects that these chemicals may cause.

FLB is a new insecticide that acts selectively on the ryanodine receptors (RyRs) of insects, and it has been widely used in recent years. Since it is relatively safe compared to other pesticide groups, it has been wide used in agriculture to control insects. Therefore, in our study, to evaluate the hazard of the FLB exposure, the behavioral toxicity of FLB on *D. melanogaster*, induced DNA damage response in two different test organisms and cytotoxicity in the *Allium* test, as well as its DNA and RyR binding strength (via molecular docking) were investigated.

In the *Allium* test, all of the applied concentrations of FLB significantly reduced the mean root length in a concentration-dependent manner compared to that of the control. The inhibition of root growth may be related to defects in the apical meristematic activity [26], retardation of cell elongation during differentiation [27] or the loss of the cell wall during differentiation. Briefly, FLB showed a cytotoxic effect by statistically significantly decreasing the root growth and MI, as well as a genotoxic effect by statistically significantly increasing the number of CAs. These decreases in MI were found to be statistically significant, depending on both the concentration and time. The significant reduction of the MI could be due to either cell cycle disruption, such as the blockade of the G1 phase, the suppression or inhibition of DNA synthesis in the S phase [28,29] or the blockage of the G phase [30]. The decrease in the MI can also be caused by abnormal changes (deviations) in the duration of the mitotic phases. Disrupted ana-telophase (Figure 3e) and chromosome laggards (Figure 3f) could be the result of the inability of the chromosomes to fully move to the opposite poles, which may be due to the dysfunction of the spindle fibers. The dysfunction in the spindle fibers may be caused by structural deformation, which is due to the disruption of the microtubules [31,32,33]. Stickiness, which imposes irreversible structural damage to the chromosomes (Figure 3g), can be caused by DNA–DNA or DNA–protein cross-links. Stickiness is likely due to depolymerization of DNA, the partial dissolution of nucleoproteins, the breakage and displacement of basic folded fiber units of chromatids and the stripping of the protein complexed with the DNA in the chromosomes [34,35,36]. The anaphase bridge (Figure 3h), which occurs as a result of the clastogenic effects of chemicals [30,37], may result from the breakage or fusion of chromosomes, unequal sister chromatid exchange, dicentric chromosomes or defects in the activation of replication enzymes or stickiness. Polyploidy (Figure 3i), on the other hand, may lead to difficulties in phragmoplast formation as a result of the disruption of the normal functioning of the cytokinetic apparatus [38].

The *Allium* test system continues to be successfully used for a comprehensive mutagenic evaluation of different chemical compounds, phytochemicals, nanomaterials and different environmental samples, as fast and reliable results are obtained with this test [14].

The comet test is a very common method that can be used to identify DNA damage in different cell types belonging to different organisms. The comet assay results showed significantly higher DNA damage rate at all of the FLB concentrations compared to those of the negative control. FLB is an insecticide included in the diamide group, and diamides have been used more extensively in recent years than other pesticide groups have due to their efficiency and selectivity [39]. There are studies showing that exposure to FLB results in the induction of apoptosis [40], oxidative stress and DNA damage [41,42]. It is known that cells are prone to damage due to increased ROS production during exposure to chemical agents. Exposure to FLB may result in lots of ROS production due to the inhibition of the activities of free radical scavengers and antioxidants.

Behavioral tests are widely used in the toxicological evaluation of xenobiotics [43,44]. In addition to being sensitive, specific and reliable for detecting the effects of chemicals, data from behavioral tests can also be useful for making regulatory decisions and mechanistic assessments in basic research. In addition, behavioral changes are one of the important research areas used in toxicity assessments, as they are the initial measurable effect of chemical exposure. This is because the behavioral responses can be determined in vivo before the clinical symptoms or structural lesions appear as a result of exposure. In particular, it is very important to note that the behavioral changes reflect the general body functioning of the organism, and they do not aim to be used a sensitive or better measure [45]. In our study of behavioral toxicity in *D. melanogaster*, exposure to FLB resulted in decreased locomotor function. The administration of FLB to larvae and adult flies hindered the locomotor functions, depicting a possible negative alteration in the nervous system, which plays an important role in the control of locomotor function in organisms [23]. The results obtained in the pupal position experiment with FLB show us that there may be an interaction between this substance and the DNA. The height of the pupa is a gravity-dependent response, and it can be viewed as a complex trait that is determined by a variety of other simple behaviors [46]. Natural variation in the pupal site selection may result from the genes and the environment, as well as gene-–environment interactions [47]. According to the data we obtained in the larval crawling assay, a decrease in larval movement was observed for the samples due to the increase in the concentration of the FLB. The reduction of larval crawling in the 1 and 0.001 mM applications compared to that in the control group was found to be statistically significant. Kumar et al. [48] exposed *Drosophila* larvae to the pesticide Retenone and evaluated the larval creep activity and pattern. In parallel with our study, they found a decrease in the larval crawling activity. It has been suggested that this may be due to the fact that dopamine, which plays a regulatory role in the neuronal networks that control locomotor activity in insects, shows a gradual and permanent decrease in its activity upon exposure to Retenone [49]. In our study, larval weight reduction was found to be statistically significant between the control group and the 1 mM FLB-exposed group. Kumar et al. [48] found a decrease in the larval weight, which is similar to our study, with an increasing ROS concentration at the end of the application period. Kumar et al. [48] stated that although the food consumption value is the same, the reason for the decrease in the larval weight in the individuals exposed to ROS could be due to increased rate of cell death. In that study, the researchers showed that the exposure to ROS caused tissue damage through the disruption of cells.

The comet test is one of the most promising methods for detecting the genotoxic potential of chemicals, as it is simple, rapid, specific and sensitive, and it requires only a small amount of the sample [49,50]. In the comet assay, an increase in the rate of DNA damage due to the FLB treatment was observed depending on the increase in the concentration. The increase in the DNA damage scores between the control group and the 1 mM FLB-treated group was statistically significant. When Xiuyuan et al. [51] evaluated the effects of 5 and 10 mg/kg concentrations of FLB on *Eisenia fetida* with the comet assay, they found that this substance posed a high risk in terms of DNA damage. The occurrence of DNA damage can be explained by an increase in the activities of both the free radicals and ROS during chemical processes. Other studies have suggested that as a result of exposure to environmental pollutants that trigger the formation of both free radicals and ROS, the antioxidant systems may not function properly, resulting in oxidative DNA modification [52,53]. FLB has been reported to significantly increase the rate of micronucleus formation, the DNA shearing pattern in the agarose gel and DNA fragmentation (comet assay) in rat splenocytes compared to those in the control group [25]. This reported capacity of FLB to induce potent DNA damage is consistent with our docking experiments. FLB binds the DNA molecule with a much higher affinity (ΔG_best_ = −7.25 kcal/mol) than the known mutagens MMS and NaN_3_ do (ΔG_best_ = −4.07 kcal/mol and −4.13 kcal/mol, respectively) (Table 2). Additionally, in a similar way to these mutagens, FLB recognizes the DNA via the minor groove and can bind with nucleotides on both of the strands of the macromolecule (Figure 9). Thus, in this way, FLB may cause the collapse of the replication fork in these regions during DNA replication and, therefore, double-stranded breaks in newly synthesized DNA in correlation with the frequency of its DNA binding.

RyRs are large calcium-release channels that are located in the membranes of the sarcoplasmic reticulum, and they are responsible for the intracellular Ca^+^ release that triggers muscle contraction [54,55]. There are three identified isoforms of RyRs in mammals: RyR1 of the skeletal muscle, RyR2 of the cardiac muscle and the more ubiquitous RyR3 of the brain tissue [54,55]. In insects, there is only one isoform of RyR, and it is expressed in the muscular and nervous system [55]. Although the exact binding site of FLB, a diamide insecticide, on RyRs remains elusive, FLB forms strong binding complexes with these receptors, causing the receptors to be stuck in a stabilized ‘open conformational’ state, triggering the intense release of Ca^+^ ions from the sarcoplasmic reticulum to the cytoplasm and leading to severe cytotoxicity coupled with muscle contraction and insect mortality [24,56,57].

In our docking study, FLB was found to form an energetically highly favorable complex with the RyR *Repeat34* domain (insect-specific druggable pocket) (Table 3; Figure 10). This type of intermolecular interaction is in line with the results of our larval crawling test which was performed on *D. melanogaster*. Gradually increasing the concentrations of FLB may block all of the RyR receptors in the muscular system, reducing the crawling speed (mm/s) in the *Drosophila* larvae statistically significantly (especially at 0.1 and 1 mM concentrations) compared to that of the control. This observed result shows that FLB disturbs Ca+ homeostasis in the muscles by blocking the RyR receptors in the ˈopen conformationˈ in *D. melanogaster* larvae. Additionally, since larval crawling is a locomotion that is controlled by the brain, it is highly possible that FLB could produce a similar stabilizing effect on the RyR receptors of *Drosophila* nerve cells. In brief, because the possible target receptors of FLB in animal organisms are more numerous due to the absence of RyR receptors in plants, it could be inferred that the resulting toxic response upon FLB exposure in these organisms is more potent than it is in plants, especially at higher concentrations.

## 4. Materials and Methods

### 4.1. Allium Test

Takumı (20% Flubendiamide) was purchased from Hektaş, Adana, Turkey. The following chemicals were purchased from Sigma-Aldrich, Munich, Germany: Sodium Hydroxide, Ethidium Bromide, Di-Sodium Salt of Ethylene Diamine Tetra Acetic Acid (EDTA), Trizma Base, Sodium Chloride, Hydrochloric Acid, Disodium Hydrogen Phosphate, MMS (CAS Number 67-27-3), Low Melting Point Agarose (LMPA), Potassium Phosphate Monobasic, Magnesium Chloride Hexahydrate, Potassium Chloride, Triton X-100, Normal Melting Point Agarose (NMPA), Basic Fuchsin, and Trizma Hydrochloride.

The *A. cepa* root growth inhibition test was performed as described by Fiskesjö [58], with modifications as suggested by Cigerci et al. [59]. In this assay, the *A. cepa* bulbs (diameter 25–30 mm, untreated) were used as the test material. After the bulbs of equal size were rooted in distilled water for 24 h, five bulbs with the same root length were transferred to the control and application solutions, each containing predetermined test concentrations of FLB. To determine these test concentrations, the EC_50_ concentration was determined prior to the experiments, using five different FLB concentrations: 500, 750, 1000, 1250 and 1500 mg/L. The concentration value of FLB (EC_50_), which reduced the growth of the roots by 50% compared to that which occurred in the negative control group, was determined as 1250 mg/L and 1/2 × EC_50_, EC_50_, and 2 × EC_50_ of the determined EC_50_ were used. Thus, the roots were treated with 625, 1250 and 2500 mg/L concentrations of FLB for 24, 48, 72 and 96 h. The FLB-exposed roots obtained at the end of the experiment were dyed with Feulgen reagent, and slides were prepared for microscopic examination. For the MI, at least 5.000 cells progressing through the different stages of mitosis per concentration were counted, and the results are expressed as percentage (%) values.

### 4.2. Drosophila Strain and Chemical Substances

The *D. melanogaster* samples used in the experiments were obtained from the Toxicology Laboratory of Biology Department, Akdeniz University, Antalya, Turkey. The colonies were kept in the laboratory at 60% relative humidity, 25 ± 1 °C temperature and a 12:12 (Light:Darkness) photoperiod. The preparation of the test solutions was performed via the dissolution of these technical substances in distilled water.

In all of the experiments, 8-hour-old eggs were collected from the *Drosophila* Oregon R^+^ line. Three distinct time applications (48, 72 and 96 h) and four different FLB concentrations (0.001, 0.01, 0.1 and 1 mM) were used to expose the collected eggs. The exposure was carried out by transferring the 72 ± 4 h larvae to the nutrient medium which was formed by wetting the *Drosophila* instant medium, approximately 4.5 g of which was dry, with 9 mL of different concentrations of FLB. The experiments were performed with 3 replications for each concentration. In addition, the distilled water used in the preparation of the test concentrations was established as a negative control group in all of the experimental groups.

#### 4.2.1. Behavioral Experiments

##### Pupa Formation Success and Pupal Position

The individuals in the application tubes containing different concentrations of FLB were fed with the medium until the larval period began. The individuals emerging from the pupa in the same tubes were recorded, and the success of hatching was calculated as a percentage using the formula: (number of adults/50) × 100% [60].

The pupal position measurement experiment was performed according to Fauzi et al. [44]. The results were determined as percentages according to the positions of the pupae. Exposure was maintained until the end of the pupal formation.

A 50 mL falcon tube was used as the application tube, and it was divided into 4 different zones at equal intervals (1.3 cm) from the food surface. At the end of the application period, the heights of the pupae formed by the larvae were determined. A total of 4 regions were marked as D, C, B and A, respectively, from the food surface upwards. The pupal position measurement experiment was carried out with 3 repetitions.

##### Larval Weight

After 24 h of exposure, the larvae in each treatment group were collected under tap water using a fine-mesh strainer for the larval weight measurement, and 10 larvae were weighed on a precision balance for each application, and their weight was measured. The experiment was carried out with 3 repetitions.

##### Crawling

Petri dishes covered with 2% agar were used for the measurement of larval movement, and the distance traveled by larvae that were placed in the middle of the Petri dish for 1 min was measured using graph paper. Thirty larval movements were measured for each concentration, and the experiment was carried out in triplicate. As a result of the experiment, the statistical evaluation was performed by taking the average of 3 repeated measurements for each concentration [61].

### 4.3. Comet Assay

The alkaline comet test was performed with the root meristem cells of *A. cepa* and *D. melanogaster* larvae that were exposed to different concentrations of FLB, with a modification of the protocol of Tice et al. [62]. After the test material was applied to the *A. cepa* stem cells, the cells’ nuclei were isolated with 500 µL of cold Tris-MgCl_2_ buffer (4 mM MgCl_2_–6H_2_O; 0.2 M Tris, 0.5% *w*/*v* Triton X-100, pH 7.5). The nuclear suspension (50 µL) was mixed with 1.5% low melting point agarose (LMA) (50 µL) and spread onto the slides coated with normal melting point agarose (NMA) (1%).

The eggs were collected for 8 h in fresh food from *Drosophila* Oregon R+ lines that were grown in a sufficient quantity. The collected eggs were exposed to different concentrations of FLB when they reached the 72 ± 4 h larval stage (3rd larval stage). The FLB exposure was carried out by transferring the larvae to 4.5 g of *Drosophila* ready-made standard broth that was soaked with 9 mL of FLB solutions at different concentrations, and the exposure was carried out for 24 h in an environment of 25 ± 1 °C and 60% relative humidity. The hemocytes were collected from *Drosophila* larvae according to the method of Irving et al. [63]. Forty to sixty larvae were selected for each application, and the hemocytes were collected in phosphate-buffered saline (PBS) solution containing 0.07% phenylthiourea using two fine-tipped forceps under a stereo microscope. After the hemocyte isolation, cold PBS (20 μL) was added to the pellet that was obtained by centrifugation at 300× *g* (10 min + 4 °C). Then, the hemocytes were mixed with 80 µL of LMA (0.75%) and spread onto the slides, which were coated with 100 µL of NMA (1%) the day before and covered with a coverslip. At the end of the preparation, these coverslips were removed, and the slides were placed in a fresh lysis solution (2.5 M NaCl, 10 mM Tris, 100 mM Na_2_EDTA, 1% Triton X-100 and 1% N-lauryl sarcosinate, pH 10) in the dark at +4 °C for 1 h. The next steps of the comet experiment were performed in the same way for both *A. cepa* and *D. melanogaster*. After the lysis step, the slides were placed in a cold electrophoresis buffer (1 mM Na_2_EDTA and 300 mM NaOH, pH > 13) and kept for 30 min, and then, the electrophoresis were run in the same buffer for 30 min at 25 V 300 mA. After the electrophoresis, the slides removed from the buffer solution were kept in 400 mM Tris solution (pH 7.5) for 5 min, and the same procedure was repeated 2 more times, which was followed by neutralization. Finally, the slides were stained with 50 µL of EtBr (60 µg/mL) and kept at +4 °C for 20 min. Fifty comets per slide were scored visually using a fluorescence microscope (Olympus BX30). The nuclear damage images were scored as belonging to one of five different classes: from 0 (no damage) to 4 (full damage).

### 4.4. Molecular Docking Studies

In this study, molecular docking was carried out in order to explain, at the molecular scale, the experimental findings obtained from the genotoxicity and behavioral impact tests of FLB and to shed light on the mechanistic nature of these adverse effects induced in the eukaryotic test organisms, *A. cepa* and *D. melanogaster*. To model the intermolecular interactions of FLB with the DNA, the crystallographic structure of a synthetic DNA dodecamer (PDB ID: 1BNA, resolution: 1.90 Å) that was downloaded from the RCSB PDB database [64] was used in the docking simulations [64]. Regarding the elucidation of the mechanisms of the behavioral effects of FLB, the following approach was followed: FLB exerts its lethal effect in insects by binding to RyR. Upon binding with the RyR receptors, it stabilizes the receptor in the ‘open conformation’, leading to its activation, and ultimately, causing muscle paralysis and death [55,56]. However, different isoforms of RyR are found not only in skeletal muscles, but they are also found in cardiac muscles and the brain [65]. Furthermore, insect RyRs are also expressed in the central nervous system [56]. Therefore, we envisaged that RyR is a suitable protein target for elucidating the molecular mechanisms of the locomotion-disrupting effects of FLB in *D. melanogaster*. The binding sites of diamide type insecticides, including FLB, on RyRs have still not been fully elucidated. However, a recent experimental study showed that a unique glycerol binding pocket in the RyR *Repeat34* domain of diamondback moth (DBM) modulates the structure and function of RyR, therefore, this region (RyR *Repeat34* domain) is proposed to be a druggable pocket in the insect-specific pesticide design [56]. In this context, the crystal structure of the DBM RyR *Repeat34* domain [66] (PDB ID: 6J6P, resolution: 1.53 Å) was used as the second target receptor in the molecular docking study. On the other hand, the 3D structure of methyl methane sulfonate (MMS) and sodium azide (NaN3) were downloaded from PubChem database in the PDB format.

#### 4.4.1. Protein and Ligand Preparation

Before initiating the docking simulations, the receptor molecules (DNA and DBM RyR *Repeat34* domain) were cleaned by removing the water molecules, inhibitors and non-interacting ions using the Discovery Studio Visualizer v16 program [67]. The missing atoms in the amino acid side chains of the DBM RyR *Repeat34* domain were added using the AutoDockTools (ADT) 1.5.6 interface [68]. Then, the geometry optimization of the DBM RyR *Repeat34* domain was performed using CHARMM22_PROT force field implemented in the Vega ZZ software (3.2.2.21) via the NAMD (nanoscale molecular dynamics) module [69]. After the geometry optimization was completed, the 3D protein structure corresponding to the last minimization step was saved in the PDB format as the lowest energy conformer. On the other hand, the 3D structure (atomic coordinates) of the FLB molecule used as a ligand in molecular docking was downloaded from the PubChem database in the SDFformat, the energy minimization was performed with UFF force field in Avogadro program, and it was saved in the PDB format [70]. The experimental positive controls MMS and NaN_3_ which were used in the experiments with *A. cepa* and *D. melanogaster*, respectively, as well as the glycerol (bound native ligand of DBM RyR *Repeat34* domain), were also optimized using the UFF force field and saved in the PDB format. Finally, the Open Babel GUI [71] and AutoDock Tools 1.5.6 [68] were used, respectively, to convert the ligands (FLB, MMS, NaN3 and glycerol) and receptor structures into the PDBQT format prior to docking.

#### 4.4.2. Molecular Docking

In this study, the latest version of AutoDock Vina (1.2.0) was used to dock FLB against the DNA and protein receptors [72].

In the docking studies with the B-DNA dodecamer and the DBM RyR *Repeat34* domain, the polar hydrogen atoms in the receptors and ligands (FLB, MMS, NaN3 and glycerol) were retained, however, non-polar hydrogens were merged using AutoDock Tools 1.5.6. Kollman charges were assigned to the receptors, whereas Gasteiger charges were assigned to the ligands. In the docking simulations, the rotatable bonds of the ligands were allowed to rotate freely (flexible ligand). The grid box size for DNA was set as 60 × 60 × 126 Å points (x: 7.03; y:22.93; z: 28.25), whereas for the DBM RyR *Repeat34* domain, it was set as 40 × 40 × 40 Å points (x: 21.72; y:11.74; z: −20.64). The dimensions of the grid box have been determined to allow the FLB to easily interact with the entire molecular surface of the DNA and the catalytic amino acid residues in the glycerol-binding pocket of the RyR *Repeat34* domain. The catalytic residues (glycerol-binding site) of the DBM RyR *Repeat34* domain were determined from the literature [56]. In addition, the binding affinity values (ΔG°: kcal/mol) obtained from the DNA-MMS, DNA-NaN_3_ and RyR-glycerol dockings were used as the control groups for comparison between the DNA-FLB and protein-FLB dockings, respectively.

The number of docking runs was set as ‘20’, and the exhaustiveness was set to ‘500’ in the dockings of FLB (as well as the positive controls) against the two target receptors. Following the docking, all of the potential binding conformations of FLB were clustered through AutoDock Vina 1.2.0 and ranked based on the binding affinity of the ligand conformation which showed the most favorable (most negative) binding free energy (ΔG°; kcal/mol) against each target receptors. The top-ranked binding poses of FLB among the different poses against each of the target receptors were visualized and qualitatively analyzed using the Discovery Studio Visualizer v16 program [67].

### 4.5. Statistical Analyzes

The root length, MI and mitotic phases, CAs and DNA damage data, which are given as the arithmetic mean ± standard deviation for the *Allium* test, were evaluated with the Duncan test using SPSS, ver. 23.0. The statistical significance level was accepted as *p* ≤ 0.05. The dose–response and time–response relationships were evaluated through the Pearson correlation at *p* ≤ 0.01.

The statistical analyzes of the results obtained for *Drosophila* were evaluated using the SPSS 23 Package One-way ANOVA program. The larval weight, pupa formation, pupa formation success and crawling were evaluated with the Kruskal–Wallis test. A posteriori analysis was performed with the Dunn–Bonferroni test.

## 5. Conclusions

This comprehensive experimental and computational study showed that FLB may pose potential risks for other non-target organisms, including humans, as it exerts its cytotoxic and genotoxic effects on two different eukaryotic organisms. The molecular docking studies have shown that FLB binds energetically and highly favorably with the DNA molecule, and it also forms a strong binding complex with the *Repeat34* domain of DBM RyR (which is also present in *D. melanogaster* muscle and neurons), which may indicate that the DNA-damaging and larval crawling-disrupting effects of FLB could be due to its ability to form strong intermolecular complexes with these target receptors.

## Figures and Tables

**Table 1:** Effect of FLB on mitotic index and mitotic phase indices in *A. cepa* roots.

Concentration (mg/L)	CCN	MI ± SD *	Phase Index (%) ± SD *			
			Prophase	Metaphase	Anaphase	Telophase
Control: 24 h	5097	66.82 ± 0.74 a	90.58 ± 0.67 a	1.97 ± 0.26 a	2.11 ± 0.28 a	5.34 ± 0.43 a
MMS-10	5102	57.19 ± 0.61 b	88.76 ± 0.66 b	1.88 ± 0.26 ab	2.92 ± 0.17 b	6.44 ± 0.73 b
625	5080	63.86 ± 0.52 c	89.18 ± 0.66 b	1.88 ± 0.13 ab	2.56 ± 0.17 c	6.38 ± 0.65 b
1250	5102	61.64 ± 0.45 d	89.32 ± 0.98 b	1.65 ± 0.17 bc	2.35 ± 0.26 ac	6.67 ± 0.78 b
2500	5100	60.63 ± 0.57 e	88.9 ± 0.82 b	1.59 ± 0.14 c	2.17 ± 0.17 a	7.35 ± 0.85 b
Control: 48 h	5092	68.56 ± 0.59 a	89.86 ± 0.84 a	1.86 ± 0.21 a	2.29 ± 0.32 a	5.99 ± 0.46 a
MMS-10	5105	55.38 ± 0.52 b	88.36 ± 0.43 b	1.74 ± 0.25 ab	2.94 ± 0.21 b	6.97 ± 0.64 b
625	5086	62.27 ± 0.49 c	89.77 ± 0.29 a	1.61 ± 0.2 ab	1.99 ± 0.18 c	6.63 ± 0.32 ab
1250	5079	60.86 ± 0.35 d	89.07 ± 0.61 ab	1.81 ± 0.26 ab	1.85 ± 0.2 c	7.28 ± 0.68 b
2500	5087	58.92 ± 0.81 e	89.22 ± 0.63 a	1.53 ± 0.12 b	1.8 ± 0.12 c	7.44 ± 0.69 b
Control: 72 h	5110	68.3 ± 0.66 a	90.06 ± 0.62 a	1.89 ± 0.18 a	2.32 ± 0.28 a	5.73 ± 0.42 a
MMS-10	5092	53.91 ± 0.79 b	87.8 ± 0.93 b	1.89 ± 0.27 a	2.99 ± 0.2 b	7.32 ± 0.74 bc
625	5086	59.97 ± 0.5 c	89.34 ± 0.78 ac	1.71 ± 0.18 ab	2.26 ± 0.22 a	6.7 ± 0.76 b
1250	5093	58.97 ± 0.71 d	89.31 ± 0.31 ac	1.56 ± 0.18 b	2.06 ± 0.26 a	7.06 ± 0.32 bc
2500	5118	56.94 ± 0.7 e	89.05 ± 0.29 c	1.82 ± 0.15 ab	1.61 ± 0.09 c	7.51 ± 0.39 c
Control: 96 h	5106	68.12 ± 0.5 a	90.14 ± 0.65 a	1.72 ± 0.14 a	2.21 ± 0.22 a	5.92 ± 0.53 a
MMS-10	5081	52.88 ± 0.57 b	87.72 ± 0.3 b	1.97 ± 0.22 ab	2.64 ± 0.27 b	7.66 ± 0.49 b
625	5072	58.4 ± 0.73 c	89.26 ± 0.45	2.06 ± 0.15 b	2.09 ± 0.1 ac	6.59 ± 0.42 cb
1250	5116	57.43 ± 0.31 d	88.8 ± 0.34	1.87 ± 0.16 ab	1.9 ± 0.22 c	7.43 ± 0.48 b
2500	5072	55.09 ± 0.61 e	88.84 ± 0.22	1.79 ± 0.19 a	1.93 ± 0.21 ac	7.44 ± 0.3 b

* Different letters in the same columns for each treatment time are statistically significant (*p* ≤ 0.05). CCN: Counting Cell Numbers. SD: Standard Deviation. Dose dependent: For 24 h, r = −0.925; *p* = 0.01. For 48 h, r = −0.931; *p* = 0.01. For 72 h, r = −0.892; *p* = 0.01. For 96, h r = −0.912; *p* = 0.01. Time dependent: For 625 mg/L, r = −0.969; *p* = 0.01. For 1250 mg/L, r = −0.956; *p* = 0.01. For 2500 mg/L, r = −0.959; *p* = 0.01.

**Table 2:** Docking binding free energies, binding mode and DNA nucleotide-interaction results, including bond lengths (Å), for methyl methanesulfonate (MMS), sodium azide (NaN_3_) and flubendiamide.

Compound	Receptor	ΔG_best_ (kcal/mol)	Binding Mode	Classical H Bond	Non-Classical H Bond	π-Sigma Interaction	Halogen (Fluorine)
MMS (positive control)	B-DNA	−4.07	Minor groove	Gua10 (2.12 Å, 2.33 Å), Gua16 (2.28 Å, 2.21 Å)	-	-	Gua10 (5.85 Å), Gua16 (5.45 Å)
NaN_3_ (positive control)	B-DNA	−4.13	Minor groove	Cyt9 (2.42 Å), Gua10 (2.45 Å), Gua16 (2.14 Å), 2.77 Å)	-	-	-
Flubendiamide	B-DNA	−7.25	Minor groove	Gua16 (2.50 Å, 2.70 Å), Ade17 (2.02 Å)	Cyt11 (3.45 Å, 3.55 Å, 3.70 Å), Gua12 (3.30 Å, 3.44 Å), Ade17 (3.43 Å, 3.46 Å)	Ade18 (3.56 Å)	Cyt11(3.02 Å, 3.59 Å), Cyt15 (3.25 Å)

MMS: methyl methanesulfonate–positive control; NaN_3_: sodium azide–positive control; ΔG_best_: the most favorable binding free energy (kcal/mol).

**Table 3:** Docking binding free energies and diamondback moth (DBM) ryanodine receptor *Repeat34* domain-interaction results for glycerol and flubendiamide in the glycerol-binding pocket.

Compound	Molecular Weight (g/mol)	Receptor	ΔG_best_ (kcal/mol)	Classical H Bond	Non-Classical H Bond	Alkyl, π-Sigma/ π-Alkyl Interaction	Electrostatic Interaction	Halogen (Fluorine)
Glycerol (native ligand)	92.09	RyR *Repeat34* domain *	−3.32	Asp3029 (2.10 Å)	-	-	-	-
Flubendiamide	682.40	RyR *Repeat34* domain	−8.23	Arg3032 (2.35 Å)	Ala2992 (3.02 Å), Lys2999 (3.11 Å)	Lys3028 (3.49 Å), Ala2992 (4.07 Å), Arg2908 (4.79 Å), Pro2912 (3.95 Å, 4.49 Å), Ile2995 (4.13 Å, 4.97 Å), Pro2912 (5.02 Å)	Lys3028 (4.12 Å), Asp3029 (4.84 Å)	Glu2911 (3.61 Å), Pro2912 (3.22 Å), Ala2992 (2.95 Å)

* (PDB ID: 6J6P): Diamondback moth (DBM) ryanodine receptor (RyR) *Repeat34* domain; ΔG_best_: most favorable binding free energy (kcal/mol). The underlined amino acid residue (Asp3029) depicts that flubendiamide can bind to this same residue to which the native ligand, glycerol, is bound in docking simulations, which proves the success of the docking conformational sampling algorithm.

## Data Availability

Not applicable.
